# Rescaling low‐carbon transformations: Towards a relational ontology

**DOI:** 10.1111/tran.12275

**Published:** 2018-10-07

**Authors:** Stefan Bouzarovski, Håvard Haarstad

**Affiliations:** ^1^ Department of Geography Manchester Urban Institute University of Manchester Manchester UK; ^2^ Centre for Climate and Energy Transformation Department of Geography University of Bergen Bergen Norway

**Keywords:** decarbonisation, hybrid geographies, networks, scale, urban transformations

## Abstract

Scale is an emergent theme in current scientific and policy debates on low‐carbon urban transformations. Yet notions of scale employed in such contexts are typically based on linear and hierarchical ontologies, and miss out on the long‐standing development of more nuanced conceptions of scale within Human Geography. This paper aims to advance a relational understanding of scale in the analysis and evaluation of low‐carbon urban initiatives (LCUIs). We wish to lay the path towards an innately geographical conceptualisation of low‐carbon urban transformations more generally, in which cities are not seen as rigid and passive physical containers for decarbonisation initiatives, but rather as key nodes within vibrant socio‐technical networks operating across multiple material sites. Using a case study of the transnational and translocal REACH (Reduce Energy use And Change Habits) project funded by the European Union as illustration, we argue that low‐carbon urban transformations are immanently constituted of three sets of relational processes across scale, involving (1) politicisation, (2) enrolment and (3) the hybridisation of human and material agencies.

## Abstract 2

This paper aims to advance a relational understanding of scale in the analysis and evaluation of low‐carbon urban initiatives. We wish to lay the path towards an innately geographical conceptualisation of low‐carbon urban transformations more generally, in which cities are not seen as rigid and passive physical containers for decarbonisation initiatives, but rather as key nodes within vibrant socio‐technical networks operating across multiple material sites.

## 1 INTRODUCTION

Socio‐technical interventions, experiments and initiatives aimed at reducing the energy and carbon intensity of human activities in cities are proliferating across the planet. Articulated principally in response to climate change imperatives, these undertakings – sometimes collectively described under the umbrella term low‐carbon urban initiatives (LCUIs) – are aimed at engendering low‐carbon transformations in the development and utilisation of urban infrastructural systems. They have attracted significant academic attention within a variety of disciplines, including Geography. But the relatively limited physical and material extent of LCUIs within the confines of particular cities or neighbourhoods has raised numerous questions about the extent to which they can challenge the underlying systemic drivers of unsustainable practices. How LCUIs might be “scaled up” via the replication or expansion of existing activities has therefore become a key scientific and policy challenge.

To date, geographers have been relatively silent in terms of explicitly exploring the relationship between the production and articulation of scale, on the one hand, and urban carbon reduction efforts, on the other (but see, for example, Bulkeley, Broto & Maassen, 2014). While Geography as a whole has been displaying increasing engagement with low‐carbon and energy transitions as part of an – arguable – “spatial turn” in this field (Bridge, 2018), questions of scale have rarely received detailed consideration within such debates. At the same time, the literature on low‐carbon urban transformations and scale is dominated by nested and linear understandings of the patterns that exist at different levels of spatial aggregation (van Doren et al., 2016). There is limited recognition of the non‐hierarchical ontologies of scale that have been proposed and developed by numerous geographers over a period of several decades (e.g., Marston et al., 2005). What is more, it is rarely recognised that the territorialities and materialities of scale both shape and are produced by the multiple domains of social and political life that take place in the urban fabric.

This paper is aimed at advancing a non‐hierarchical conception of scale in the analysis and evaluation of LCUIs. Building on insights from the long‐running debate on scale in Geography (Swyngedouw, 1997), we develop a perspective that highlights how successful LCUIs are able to achieve transformational change by enrolling a variety of actors operating at different levels of governance, while at the same time politicising the power relations and inequalities that underpin the production of urban space – a process of “rescaling.” In this, we wish to open the space for an innately geographical conceptualisation of low‐carbon urban transformations, in which cities are not seen as rigid and passive physical containers for decarbonisation initiatives, but rather as key nodes within vibrant socio‐technical networks operating across multiple material sites. Such an effort, we hope, can provide a geographical corrective to mainstream interpretations of sustainability transitions (Markard et al., 2012), while contributing to the rise of an enhanced sensitivity within Geography itself with regard to emergent debates on socio‐technical change in cities (Moss, 2014).

Empirically, the paper is articulated in relation to a case study of the transnational and translocal REACH (Reduce Energy use And Change Habits) project, funded by the European Union's Intelligent Energy Europe programme. REACH encompassed LCUIs with varying degrees of success, located in several cities across South‐eastern Europe. Our evidence base consists of interviews with 40 government decision‐makers, advocacy activists, academic experts, business representatives and community‐level practitioners, including three participants in the REACH project. The number of interviews – and their scope – was determined by an approach aimed at reaching the widest possible spread of relevant stakeholders, while not repeating interviews with similar types of actors. We also attended three of the project's public conferences, in addition to surveying a wide range of documentary evidence – policy papers, news reports, as well as regulatory and legal acts. This was undertaken in order to illuminate the contextual and procedural aspects of LCUIs. The material gathered in this manner was analysed interpretively, in line with the extensive ontological development of relational frameworks over a prolonged period of time, drawing from, *inter alia*, Heidegger, Simondon and Deleuze, and operationalised within Geography by authors such as Harvey (2006).

We argue that the rescaling of low‐carbon urban transformations is immanently constituted of three sets of relational processes. First, this necessarily involves processes of politicisation, expressed by the ability to challenge established power relations, ideological systems and logics of capitalist social reproduction beyond the territorial location of a given LCUI. Second, it requires enrolment: interaction, knowledge exchange and engagement with actors operating at multiple levels of governance, and involving state and non‐state organisations alike. Third, it is necessary to recognise a dynamic of hybridisation, involving the co‐constitution of human and non‐human agencies in the technical infrastructures for the provision and regulation of energy (Bridge, 2015).

The paper commences with an exploration of the manner in which issues of scale and space are treated in the low‐carbon transitions literature more generally, subsequently entering into a dialogue with existing scholarship on the “upscaling” of LCUIs. We then formulate a non‐hierarchical understanding of scale, based on geographical debates and insights into the production and articulation of scale. Using evidence from the REACH project, the paper subsequently develops a relational framework for understanding low‐carbon “rescaling” (Cohen & McCarthy, 2015), by exploring how decarbonisation is politicised and networked across urban and regional landscapes. This is followed by an analysis of the material associations and interactions that are implicated in the re‐scaling of low‐carbon initiatives – again, with the aid of vignettes from the REACH project. The paper's conclusion highlights the new research pathways unlocked by a relational ontology of scale in LCUIs, in geographical scholarship and beyond.

## 2 LOW‐CARBON URBAN TRANSITIONS IN A SPATIAL CONTEXT: A CRITICAL PERSPECTIVE

To date, LCUIs have largely been understood with the aid of frameworks derived from studies of “socio‐technical transitions, system innovations and the emergence of sustainable technologies” (Markard et al., 2012, p. 957). Within this broad vein of work, the key emphasis is on socio‐technical “systems” – a notion that can be related to the concept of “large technical systems” (Mayntz & Hughes, 1988) as well as the broader vein of “systems thinking” (Rotmans et al., 2001). While socio‐technical systems are said to combine a variety of heterogeneous elements – multiple institutions, actors, material artefacts and knowledge – spatial formations and processes have rarely entered the mix. Much of the present scholarship in this line of work can be traced to socio‐technical regime theory, evolving from a foundational paper by Rip and Kemp (1998) that sought to explain how technological innovations affect society as a whole. Drawing on ideas from Latour (1987) on how scientific and technical discoveries are brought into society as “immutable mobiles,” Rip and Kemp (1998) then conceptualise technological change as originating within protected *niches* – allegorical to Latour's laboratories.

This basic idea has subsequently spawned several different strands of social theory on change, of which Markard et al. (2012) identify four: transition management, strategic niche management, the multilevel perspective (MLP) on sustainability transitions and technological innovation systems. Within the debates of concern in this paper, it is perhaps Geels’ (2011) formulation of the MLP on sustainability transitions that has gained the most currency. In one contribution (Geels, 2013), he conceptualises cities as sites of niche innovations, and describes urban‐led transitions as occurring when urban innovation communities join together in broader networks eventually affecting the national scale. Again, the basic ontology is one where innovations grow out of spatially limited seedbeds, and the most relevant case studies are techno‐scientific.

As several authors have pointed out, from a geographical perspective this conceptualisation of transitions runs into problems (Bridge et al., 2013). First, the language of niche, regime and landscape – seemingly geographical metaphors – translates uneasily into geographical scales. Relational approaches from Economic Geography have been mobilised in this context to develop a constructive critique of the MLP and propose alternatives (Coenen et al., 2012). Second, the idea that innovations occur in protected niches seems problematic when the innovations we are talking about are social and political, rather than technological and scientific. Yet much of thinking on transformations towards sustainable, low‐carbon futures is animated by the idea of transitions as originating in innovations created in local, protected spaces (Brown & Vergragt, 2008) that subsequently interact with broader socio‐technical regimes.

Focusing on contributions on sustainability transformations in cities, it is evident that some problematic assumptions about “upscaling” are derived – implicitly or explicitly – from the theory of socio‐technical transitions. Dynamics of change in cities are now predominantly framed through a combination of concepts such as socio‐technical regimes, local innovation, protected spaces, labs and experimentation (Haarstad, 2016a). For example, in the work on Urban Transition Labs (UTLs) (Nevens et al., 2013), these formations are conceived as “incubators of change.” Mainstream conceptualisations of UTLs are, like much of the current theorising, explicitly set in the sustainability transitions literature. From there they take the idea that “pilot projects can act as seeds of transformation in a policy context when their benefits and outcomes are well shared and communicated” (p. 112). But the emphasis of the approach is to “find and safeguard a certain protected comfort zone” in which “frontrunners” engage in envisioning and backcasting. UTLs remain within an ontology of labs as a protected niche or incubator for new practices and ideas – less attention is paid to the operationalisation of moving the enlightened ideas out of the lab, or how complex translocal relationships support that process. Even more recent theorising on sustainable urban transformations and transitions operates within the same heuristic (Ehnert et al., 2018; Ernst et al., 2016), understanding cities as distinct nodes in the interactions between niches, regimes and landscapes (also see de Haan & Rotmans, 2011).

Similarly, we find that related paradigms of Urban Living Labs (ULLs) and experimentation are embedded with the niche ontology, and fail to engage geographical insights on scale. ULLs have become a favoured approach by policy‐makers and research funders, in the European Union especially. They purport to enable the application and testing of technical and social interventions in specific contexts, and in turn provide platforms for rescaling and replication. ULLs use an experimental logic and, by empiricising and monitoring the urban landscape, can provide data to be fed into subsequent policy planning or social learning (Evans et al., 2015). Karvonen and van Heur (2014) trace the current notion of the urban living lab to science and technology studies (STS), and particularly Latour's work on how the findings generated in particular laboratories are transformed into widely accepted social “facts.” Experimentation and innovation in particular contexts are assumed to be translatable across space and scale, to achieve a transformation of socio‐technical systems.

Correspondingly, experimentation has become a dominant mode of thinking about low‐carbon activities in cities. It shares some obvious affinities with the ULL approach, through its ontological orientation towards the idea that learning and insights emerge in niches embedded in socio‐technical regimes (Bai et al., 2010). Evans (2011) describes the experimentation technique as attempting to achieve radical social and technical transition by testing out technologies and interventions in visible ways – they become the practical dimension of governance of the rather complex and intractable problem of climate change adaptation. Ergo, experiments represent an opportunity for researchers, policy‐makers and stakeholders to “ground” their activities and to work directly to “cure” urban problems: “the city becomes a laboratory‐clinic, in which cures are both developed *and* practised” (Evans, 2011, p. 266).

It is easy to understand why urban living labs have caught on – they seem to respond to a demand for localised, concrete and immediate solutions, and open for engaging people in co‐production of urban futures. They can position researchers at the pivotal point in transformation processes, placing them in critical positions to monitor solutions and facilitate learning. However, it is less clear how experimentally generated social learning and policy‐experience feed into wider processes of change beyond the urban context (Marvin et al., 2018). Indeed, a distinctive vein of critical contributions in this area has argued that practices of experimentation in the sustainability domain enact novel forms of governance, while aligning “with neoliberal rationalities insofar as they protect and further extend market relations” (Evans, 2016, p. 440). Similarly, Caprotti and Cowley stress that a “critical engagement with the notion of urban experimentation is now not only useful, but a necessity” (2016, p. 1), identifying areas such as crisis discourse, boundaries and non‐human agency as some of the critical issues in this context.

Living labs and urban experimentation approaches offer a welcome focus on concrete initiatives and situated governance strategies. However, the specific mechanisms for rescaling low‐carbon action remain outside the explicit focus of this literature. In the low‐carbon domain, there is a limited understanding of the trans‐local political coalitions and displacements that enable place‐based interventions to be replicated elsewhere, while enacting deeper transformations of state policy. Embedded within the ontology of niches and socio‐technical regimes, ULL and experimentation theorising is principally orientated towards elucidating the socio‐infrastructural environments that allow for technological and scientific innovation. Given other geographical insights about the relationality of place‐making (Robertson, 2018), it is doubtful that the idea of protected niches accurately describes the way learning and experimentation occurs. Also, it remains unclear to what extent niche–regime interactions address the underlying political conditions of urban carbon reduction efforts: how are theories about interactions between innovative niches and socio‐technical regimes relevant to the generalisation of social learning from urban governance experiments? We turn to these questions in the next section by paying attention to how scale is conceptualised in contributions that explicitly deal with these issues in the context of urban decarbonisation initiatives.

## 3 EXPLICIT ENGAGEMENTS WITH QUESTIONS OF SCALE AND SPACE IN THE LCUI LITERATURE

In policy arenas, “upscaling” is used almost interchangeably with “replication” – a quantitative increase in the number of times an innovation or intervention is copied in other locations (Haarstad, 2016b). The academic debate, on the other hand, has in different ways attempted to theorise upscaling as a deepening of the connection between specific interventions and the broader socio‐spatial structures that condition low‐carbon transformations. When scale is discussed, it is typically within a hierarchical conception largely interchangeable with organisational levels (Hansen & Coenen, 2015; Moloney & Horne, 2015). Recent contributions by van Doren et al. (2016) and Fuhr et al. (2018) are among the few to engage explicitly with the question of scale in the understanding and promotion of LCUIs. Starting from the premise that the municipal level is increasingly recognised as an appropriate level for addressing climate change and promoting low‐carbon urban development (p. 2), van Doren et al. (2016) argue that LCUIs cannot play an important role in global climate change mitigation efforts without being extended and applied beyond “islands of excellence.” They thus propose a “taxonomy of scaling‐up” that distinguishes between the augmentation of “means,” such as initiatives and programmes, on the one hand, and “ends” such as socio‐economic and environmental impacts, on the other.

While recognising the political‐spatial understanding of scale that has prevailed in Geography, van Doren et al. (2016) nonetheless choose to rely on the literature on upscaling grassroots organisations, as well as grassroots niches and experiments. This leads them to put forward an approach in which the pathways to “scaling up” are ordered in a linear and nested manner – suggesting that LCUIs can be expanded via spatial growth such as diffusion or replication, or via vertical methods such as institutionalisation, mainstreaming and translation (Bai et al., 2010). Even if there is reference to practices of structural learning and policy networks within this vein of work, there is still an emphasis on increasing the market demand for LCUIs, while situating institutional change with the neat boundaries of different political jurisdictions – moving from the local to the global.

Sustainability transitions thinking also provides the leitmotif in Hamilton et al.'s (2014) exploration of the upscaling of community‐scale carbon action at the regional (i.e., county) level in Oxfordshire, England. This contribution advances the strategic niche management approach, in which the plausible visions, social networks and learning processes underpinning “market niches” take centre stage. Another key pillar of the theoretical frameworks adopted by the authors is provided by the “middle out” framework (Janda & Parag, 2013), which converts the transition's management classification of “landscapes,” “niches” and “regimes” into a linear hierarchy involving “top,” “middle” and “bottom” actors. Despite the lack of an explicit theorisation of the spatiality of such agents in low‐carbon and energy transformations, the paradigm is based on a nested conception of scale. This is illustrated by the understanding of policy and government as upstream activities, and the relegation of energy consumption to the “downstream domain.”

Strategic niche management is also used as an organising framework in Seyfang and Haxeltine's (2012) seminal exploration of the bottom‐up “Transition Towns” movement's contribution to low‐carbon transitions in the UK. Upscaling receives explicit treatment in this contribution, being primarily seen as a route of innovation diffusion, and a means of “expanding the movement beyond committed environmentalists” rather than a question of “geographical spread” (p. 389). Moloney and Horne (2015) provide a similarly nuanced account of the relationship between urban transformations and local experimentation in the context of climate mitigation measures. They use a combination of multi‐level governance (MLG) and MLP approaches, while building on Hodson and Marvin's (2010) argument about cities as active spatial sites of innovation and change, where intermediary organisations may play a key role in transferring knowledge and power. In their approach, however, governance and spatial scale are conflated in a heuristic that recognises only the “national,” “state,” “region,” “local” and “community group” levels in the context of MLG and LCUIs. Of note in this context is also Middlemiss and Parish's (2010) contribution about the role of grassroots initiatives in creating low‐carbon communities. The two authors emphasise that such forms of action can draw on “their community to break current social boundaries, by creating new capacity for social change” (p. 7566). More broadly, the MLG literature has made a significant contribution to understandings of how climate change‐focused urban interventions are organised and steered via transnational networks of local actors (Kern & Bulkeley, 2009) as well as the variegated forms of power resources, capacities and structures exercised in this context (Marquardt, 2017).

Juxtaposing such contributions against the more conventional literature on upscaling LCUIs leads us towards highlighting the need for conceptualising politically disruptive low‐carbon action and development, in which dissensual political work (Rancière, 2015) undermines the foundational political economies of fossil fuel capitalism. In Hamilton et al. (2014), for example, the evolution of low‐carbon action involves a movement from “local community sustainability groups” via “takeoff” and “replication” to “mainstreaming” across the city and country. How the observed expansion of LCUIs may have interacted with the spatial and political forces that constitute the socio‐material landscapes of energy and “carbon control” (While et al., 2010) is outside of the authors’ purview. Low‐carbon interventions are rendered as discrete entities whose sole objective is to proliferate in a linear manner against the background of a politically passive territorial setting.

## 4 LOW‐CARBON URBAN TRANSFORMATIONS AND SCALE: TOWARDS A RELATIONAL ONTOLOGY

Efforts to overcome the linear and hierarchical scalar ontologies that underpin mainstream LCUI debates can benefit from the long‐standing development of the concept of scale in Human Geography. Since the 1990s, when the scale debate perhaps hit its first peak in interest, human geographers have thought about and debated what scales are, how they come to be and how they shape society (Brenner, 1998). A key thread running through most work in Human Geography is that what happens at a particular scale can only be understood in relation to other scales and in relation to the socio‐spatial processes that produce them (Howitt, 1998). For example, “local” social movements are not seen to operate in some isolated protected space like a niche – to the contrary, networked and cross‐scalar relationships are critical to how local movements are constituted, how they mobilise and what resources they draw on (Haarstad & Fløysand, 2007).

A relational understanding of scale would position ULLs and experimentation approaches as interconnected arenas produced and constructed by social and institutional practice. For the purposes of our discussion, this has several implications for how we conceive scale and rescaling. For one, scale cannot simply be replaced by “level,” a narrower term that is typically used to denote a point in a vertical hierarchy of institutional arrangements. Scale embodies political, social, economic and discursive processes that cannot be reduced to a particular governance institution. For example, the national “level” draws attention to the national government, while the national “scale” involves the political, economic and cultural processes that produce national institutions, “national culture,” etc. These are socially enacted and re‐enacted, in dialogue and tension with local communities and other nations. Second, scales are not discrete units within clearly delimited spatial or territorial confines. Rather, in Herod's formulation, they are “deeply interconnected as part of a continuum of social existence and praxis, with each scale shaping others” (2011, p. xv). Scales intersect and influence one another to such an extent that social practice can hardly be understood without considering cross‐scalar relations. It is tempting to ask: can we find an ULL that has not been inspired, influenced or enabled by contemporary national and transnational discourses on urban sustainability, low‐carbon transitions and stakeholder involvement?

Finally, scales should not be assumed to easily find their place in a nested hierarchy, like Russian dolls. In one sense the global scale is intuitively “higher” than the national scale, which is above the local, etc. – thinking heuristically about scale in this way may be necessary to put together an analytical narrative. But it is critical to avoid hierarchical assumptions embedded in much of the non‐relational thinking on scale. As Gibson‐Graham (2006) showed in their critique of the discourse on globalisation as an all‐powerful, masculine, dominant force that eviscerated localities and place, there is a tendency to assume that the higher the scale, the more important and consequential the processes are. Non‐relational thinking often sees scales as discrete units that are nested on top of one another in a linear fashion, like steps on a ladder. In contrast, relational thinking on scale sees scales as produced through the relationships that actors engage and negotiate from the contexts in which they are embedded. The urban scale, for instance, is always connected in different ways to other cities and scales – the national and the global is always constituted within the urban, and emanate from specific actors and institutions that can be observed first‐hand in cities. Even though the urban scale is materialised and territorialised in particular ways, it is also open and continuously renegotiated. Discourse, power and contentious politics are central to these processes of territorialisation and negotiation around scale (Allen, 2003; MacKinnon, 2011; Massey, 2007; Swyngedouw, 2004).

Engaging a relational concept of scale opens possibilities for more nuanced analyses of the rescaling of LCUIs. In particular, it helps avoid the limitations of the notion that innovations are generated into the protected space of the niche, and instead calls attention to the constructive relationships and networks that enable these initiatives to prosper. The lessons from the literature on the spatialities of social movements and contentious politics is that significant discursive and material resources are drawn on through these cross‐scalar networks (Leitner et al., 2008). Likewise, most LCUIs are in one way or another engaged in constructive relations and networks across scale, or to use Cox's (1998) term, “spaces of engagement.” As the urban policy mobility literature illustrates, urban policies and interventions are shaped through often subtle and ephemeral influences from policy‐making circuits and networks (Temenos & McCann, 2013).

Also, we can apply more subtlety to the analysis of the scaled structures that condition the success of LCUIs. Rather than mapping socio‐technical regimes on pre‐configured regional or national scales, we can conceptualise scalar structures as complex and multiple vertical and horizontal scalar settings. These scalar structures do not have causal powers in themselves; rather they are arenas for articulating the relationships and networks that enable and constrain urban low‐carbon transformations. In an insightful contribution, Hodson and Marvin (2012) argue that, as cities and regions are integrated into wider energy systems and complex socio‐technical networks, there are multiple scales of action involved in co‐ordinating energy systems and their low‐carbon transition. Such relational thinking also means that scale alone is insufficient to explain the processes at hand; the production and negotiation of scale involves various other spatialities such as flows, connections, networks, sites, places and materiality (Leitner et al., 2008).

Ergo, foundational debates on scale have a lot to contribute to the current debate on the rescaling of low‐carbon transformations. Geography's relational theorisation of scale can potentially lead to a radical rethink of current understandings of how local low‐carbon action may travel across different spatial scales. In enrolling diverse and fluid spatialities, it opens the path for recognising interventions that are transformative and emancipatory in their efforts to challenge dominant relations and practices of political power. However, what can an enhanced theoretical sensibility towards the production and performance of scale in low‐carbon urban transformations bring to existing debates? And what might an alternative reading of the myriad material sites, levels and hierarchies through which urban decarbonisation occurs look like? While limits on space prevent a more detailed empirical elaboration of all these potential aspects of the connections between LCUIs and scale, we proceed by introducing a brief empirical illustration to bring to life the key arguments underlining our effort to further a relational perspective on rescaling.

## 5 POLITICS OF URBAN DISRUPTION AND ENROLMENT IN THE MAKING OF LOW‐CARBON TRANSFORMATIONS

A relational sensibility towards the production and experience of scale in LCUIs leads us to argue that rescaling necessarily involves processes of *politicisation*. Rescaling an urban initiative is not simply achieved by replicating or transferring a policy idea or concern into a higher arena. Low‐carbon initiatives immanently require some form of disruption of established power relations and ideological systems beyond their immediate territorial location (Grandin et al., 2018). In opposition to the logic of universal market take‐up, this requires attending to the “dissensus practices that act as living indicators of what/where urgently needs to be addressed” (Kaika, 2017, p. 1). Such a project requires “a committed and explicitly political programme for transitions” (Scrase & Smith, 2009, p. 707), recognising both the multiple coalitions that institutional and grassroots actors have to build in achieving shared goals, as well as the tensions that may arise when established understandings are disrupted. Therefore, understandings of rescaling processes must take account of the ways in which LCUIs manage to disorder, rearticulate or undermine political structures that maintain a fossil economy.

Attention to the multiple political arenas of energy transformation is evident in the scaling relations and practices embedded in the REACH project, which, as noted above, involved a complex set of low‐carbon interventions in several SEE countries: Bulgaria, Croatia, Macedonia and Slovenia. Overall, the project was aimed at addressing energy poverty at both the “practical and structural level” (Živčič et al., 2016, p. 789). Energy poverty expresses a household's inability to secure socially and materially necessitated levels of energy services in the home (Bouzarovski & Petrova, 2015). The condition, which represents a form of material deprivation, extends beyond income poverty due to the role of a combination of factors: energy‐inefficient homes, low earning levels, high energy prices, as well as the manner in which residential energy is consumed (Thomson et al., 2016). As such, it reflects wider injustices in the provision of energy, particularly with respect to the institutional underpinnings of infrastructural regulation, the societal and political recognition of vulnerable households, and the ability of affected people to access adequate forms of support (Bouzarovski et al., 2016). Energy poverty is particularly widespread in South‐eastern Europe, where infrastructural, social and economic legacies inherited from the past have combined with high rates of income deprivation and poor quality housing to push energy deprivation levels well above the European average (Bouzarovski et al., 2012).

The REACH project is one of the few initiatives to recognise both the systemic nature of the problem as well as its implications on the conduct of everyday life. The project itself grew out of a long‐standing engagement with issues of low‐carbon transformation, energy restructuring and social equity among a group of non‐governmental organisations – primarily advocacy groups and practitioners. A set of favourable political circumstances implied that these actors could generate a transnational coalition to garner support from the European Union's Intelligent Energy Programme (IEE), as well as local and national authorities. The project is thus emblematic of an LCUI that has managed to rescale itself in an effective manner: transferring innovative socio‐technical insights and knowledge across a variety of governance levels while affecting changes in strategy and behaviour among decision‐makers and households alike.

In achieving a successful rescaling process, REACH itself drew on the multi‐layered networks and methodologies developed under the auspices of two IEE‐supported projects: “ACtions in low income Households to Improve energy Efficiency through Visits and Energy diagnosis” (ACHIEVE) and “Energy‐Check for Low Income Households” (EC‐LINC). Rather than emphasising income support and short‐term assistance – conventional measures to reduce energy poverty – REACH focused its attention on undertaking energy efficiency improvements in the residential sector via overlapping communities of place and interest. However, and perhaps partly to reflect the ethos of its funder, many of the project's documents operate with the language of “reducing” energy use among low income households, even if under‐consumption of energy services (rather than raw energy arriving at the boundaries of the home) is one of the key underpinnings of energy poverty. This discourse was also present in the ACHIEVE and EC‐LINC projects. The apparent tension between discourses that tend to “blame the poor” (Dorey, 2010) for their problems, on the one hand, and the stated aim of reducing disparities in access to energy services, on the other, does not seem to have been explicitly discussed in any of these projects. But unlike EC‐LINC and ACHIEVE, REACH incorporated a clear engagement with the systemic political forces that drive energy poverty, as well as the decision‐making fora and institutional arrangements that contribute to the rise of this predicament:

We started working there with energy poverty issues, and we perceived that there are certain limitations to only hav[ing] practical approaches in abating energy poverty. We sensed that it is also necessary to have more structural approaches, like developing new national programmes, new national policies, and also on the international level. So this was one of the guiding motivations in developing REACH. (Personal communication, REACH project member, 29 November 2016)

The political work of the organisations involved in running the REACH project thus spanned a multiplicity of decision‐making sites. Politicisation strategies in this sense extended beyond the territorial confines of the urban experimentation sphere (as described in Voytenko et al., 2016) to encompass a much wider variety of governing actors, policies and frameworks. They helped introduce novel and path‐breaking insights to mainstream energy efficiency debates, by connecting the rise of infrastructurally related social inequalities to both everyday practice and the provision of infrastructural services and state decisions. Dissensus was generated by opening the space for disagreement and criticism (McClymont, 2011) across a variety of fora (e.g., by involving representatives of excluded households in high‐level policy discussions and challenging decision‐makers’ views in public meetings), while embracing “exclusions, a set of items that are not simply unsaid, unseen and unheard as such” (Boano & Kelling, 2013, p. 43) in understanding how domestic energy is consumed and experienced. Nevertheless, the use of a deficit model‐based ethos of end‐use energy reduction also reflects a clear declarative commitment to align with the wider objectives of supranational decision‐making institutions, so as to ensure their strategic support.

Thinking of the manner in which REACH was (co‐)produced and effectuated leads us to a second component of rescaling in a relational sense: it must involve what we here refer to as *enrolment*. Indeed, part of the ULL and experimentation literature has argued that the extension of sustainability practices from one territorial domain to another requires translating and bringing on board the concerns of related actors in different places and networks, and a co‐articulation of these concerns into broader and more powerful forces of change (Laakso et al., 2017; Luederitz et al., 2017). Here, REACH moved beyond the dynamics commonly identified in mainstream experimentation thinking: even if its rescaling involved new interactions, knowledge exchange and engagement with state and non‐state actors in the urban arena and beyond (also understood as “scaling up” or “scaling out” – see Laakso et al., 2017), it also represented and mobilised the micro‐practices of households and individuals through a multi‐sited network of governance and power relations.

The transferability of low‐carbon urban transformations has been linked to the emergent literature on “mobile transitions” (Affolderbach & Schulz, 2016), which pays attention to the enrolment of multiple actors into processes of change. Even if building on transition studies scholarship, this body of work insists that sustainability innovations represent opportunities for knowledge transfer via, inter alia, “relational thinking that blurs the boundaries between niches and regimes” (2016, p. 1946). Also of relevance here are efforts to develop a typology of the “transnational linkages” associated with the global expansion of “sustainability experiments” (Wieczorek et al., 2015), which highlight how different capabilities and resources are deployed towards cross‐border and multi‐scalar mobility. All these analytical inroads recognise the political and relational character of spreading knowledge, ideas and ideological premises for low‐carbon transformation, and the contingent process of bringing other actors and networks into co‐articulations around common concerns.

A key element in the success of the REACH project was the establishment of networks of co‐operation among the multiple stakeholders that were engaged with, or relevant to, the LCUIs. Alongside decision‐makers within the initiatives’ host regions and countries, as well as the European Union, these connections necessitated deep political work within the immediate spatial context of the project, including the engagement of “social care/support services, local authorities, social advisors, schools, local energy providers, building managers” (Živčič et al., 2016, p. 794). The process commenced with an effort to map local governance actors and target households, in addition to tracing the communication channels that would be established to reach them. In order to facilitate the process, project partners formed an additional framework for the transfer of “practical experience, know‐how and skills” within the consortium network. This also involved the tailoring of training materials to the specificities of local settings, and acknowledging that “creating and maintaining local networks is time consuming and it must be done regularly” (2016, p. 804). A similar effort was in place when project activities were extended beyond local context:

Within REACH we tried to work with actors who were previously already identified … and we tried to link them together into a national programme, and this was really successful because now we do have a national programme for advising energy poor households, which is bringing together basically three ministries. But we also managed to stimulate a financing programme for renovation for energy poor households; for example, now they're able to get a 100 per cent subsidy for insulation. (Personal communication, REACH project member, 29 November 2016)

In moving outside the geographical confines of the case study area, REACH activists undertook a systematic identification of relevant individuals and institutions, whose engagement was subsequently spurred via the “concerted formulation of structural solutions” (Personal communication, REACH project member, 29 November 2016). In the process, project members further politicised energy poverty by pointing, in part, to its underpinning injustices in the privatisation and regulation of the energy sector, as well as the continued political neglect of energy efficiency and affordability among low‐income households. This was achieved both through informal exchanges and public announcements – press releases, conferences and publications (see http://reach-energy.eu/publications). At the same time, project members actively reflected on the external strategic context of their actions, while deciding on the kinds of alliances that they would need to build with sympathetic actors, and the recommendations that would have to be communicated to the policy arena. All of this points to the complexities embedded in LCUI diffusion, when viewed through a lens that takes into account the hybrid socio‐technical constitution of scale. Rather than a linear, one‐way dynamic of “scaling up” (as described in van Doren et al., 2016), we observe the existence of internally messy and contradictory practices of socio‐technical multiplication, in which conflicting objectives and ideologies may remain non‐reconciled.

## 6 ENGAGEMENTS WITH THE MATERIAL: RESCALING AS A SOCIO‐TECHNICAL PROCESS

Because different LCUIs are embedded in different infrastructural and material contexts, rescaling necessarily requires various forms of material engagement with the technical infrastructures for the provision of energy, as well as an understanding of the multiple processes of measurement and standardisation (“metrology”) involved in its regulation (Bridge, 2015). Alongside the existence of relations of politicisation and enrolment, therefore, it is also possible to recognise a dynamic of *hybridisation* in the co‐constitution of human and non‐human agencies that underpin low‐carbon action. This accounts for the multiple, vibrant and ever‐evolving socio‐material entanglements and different forms of technical knowledge embedded in low‐carbon interventions, also reconfigured in processes of rescaling. In destabilising linear understandings of rescaling processes, the framework of hybridisation reflects on Barry's (2006) notion of technological zones – complex territorial spaces that do not necessarily overlap with established levels of governance but rely on common “metrological,” infrastructural or assessment practices in order to standardise socio‐technical practices within a given spatial realm.

We can also draw on metabolic theorisations of urban socio‐material flows (Heynen et al., 2006) as well as insights from the literature on maintenance and repair (Graham & Thrift, 2007), to examine how LCUIs become transformative and durable in an infrastructural sense. It becomes necessary to think about the explicit spatial formations and modalities that underpin the socio‐technical character of LCUIs – experimentation, mobility, indeterminacy. Reclaiming ground from the predominance of non‐geographical perspectives in the sustainability transitions literature offers new opportunities for understanding how the messiness that underpins production of scale is both constitutive of, and constituted by, the infrastructural reconfigurations that underpin urban low‐carbon intervention.

There are multiple strands of work that can be useful to dissect hybridisation. In the energy domain, the assemblage of “socio‐energetic nodes” (Debizet et al., 2016) has been proposed as a means of understanding how infrastructural transformations interact with urban landscapes, while introducing an explicit theorisation of geographical scale to sustainability transitions thinking. Socio‐energetic nodes are understood as material installations that process and supply various forms of energy, while interacting with a multitude of human and non‐human actants. Networked and fluid forms of socio‐technical interdependence also feature in the growing line of contributions in the broader urban energy governance vein (Rutherford & Jaglin, 2015).

Dynamics of hybridisation were embodied in various practical activities undertaken within the REACH project. They involved the delivery of free energy audits and advice, accompanied by the provision of various low‐cost devices to reduce energy and water use among a relatively large group of (mostly) urban residents in the four case study countries. In terms of advice, target households were told about existing built environment issues, such as poor insulation, inefficient heating systems, and the presence of mould. The advice itself was primarily provided on‐site, with the aid of pupils and teachers from vocational schools as well as local volunteers who were specifically trained for this purpose. The project thus required an active engagement with the infrastructural networks through which energy circulates and is consumed, and the acquisition of technical knowledge among (relative) non‐experts. This was subsequently applied to a diverse set of indoor environments. The successful implementation of the relevant decarbonisation measures thus hinged on the active hybridisation of human and non‐human agencies involved in the making of home (Bouzarovski, 2009). Evaluations of the implementation of the project highlighted the nature of “human contact” (Živčič et al., 2016, p. 804) with the targeted households, as well as “social and communication skills” (2016, p. 803) among the energy advisers as key success factors, “even if a large part of the work around the visits is technical” (2016, p. 803) involving multiple calculations and assessment of possible energy and water savings. In response to a question on whether the socio‐technical nature of energy provision was an obstacle to the implementation of these energy efficiency or advice measures, one of our interviewees stated:

Yeah we have many examples. One … that most of the heating is done by individual fuel stoves, so it's very hard to use heat saving measures, and even if you had more money and you improved the heating system, you would probably not have energy savings but instead just an improvement in quality of life. Then also there were problems with incorrect electrical wiring, so in some cases it was even dangerous to change something as simple as a light bulb because the wires were damaged at the part where you screw in the light bulb – the socket was sometimes broken. And in some cases the electrical wiring was even damaged to the extent that you could see the wires exposed. (Personal communication, REACH project member, 11 November 2016)

Engaging with the materialities of low‐carbon interventions brought to the fore the relevant actors’ ongoing and past struggles with deeper urban contingencies and legacies. In this sense, LCUIs can be seen as nexus arrangements that act as “urban junctions,” helping “mediate between different infrastructure systems, different logics of demand and supply, and between social groups with particular interests” (Späth & Rohracher, 2015, p. 275). The socio‐technical logic of urban carbon action therefore is fundamentally relational and multi‐scalar: it enrols “socio‐spatial formations, intertwining of materials and collaborative enmeshing of social nature” (Heynen, 2014, p. 599), which are subsequently stabilised into new material configurations. In the REACH project, this was most pronounced in the case of district heating networks – large networked infrastructures inherited from socialism, which link buildings, people and energy systems through the provision of centralised heat to urban households. Despite providing a key connective tissue within the energy landscape of REACH's target cities (two of the project's sites were medium‐sized cities, while others included a range of smaller towns), issues with the upkeep and cost of district heating infrastructures meant that they often acted as a key driving force of energy poverty. In order to address and overcome this challenge at the level of both everyday life and policy‐making structures across different governance sites, project actors had to engage with the material practices of energy comfort (Madsen, 2018; Shove et al., 2014), the everyday articulation of repair and maintenance (Edensor, 2011; Soaita, 2012) and competing socio‐technical visions over the pathways of energy transformation (Rutherford, 2014).

## 7 CONCLUSION

Our journey through the variegated knowledge landscapes of rescaling practices associated with LCUIs has yielded a strong emphasis on the need for developing customised understandings of the multi‐faceted spatialities of urban transformations in a socio‐environmental context. In seeking to destabilise the nested hierarchies of scale that currently permeate many accounts of urban decarbonisation, we have argued that the rich theoretical understanding of scale – articulated by human geographers in particular – is not reflected in mainstream sustainability transitions debates on the diffusion and expansion of low‐carbon action in cities. While the urban experimentation and living labs literature has introduced a significant amount of conceptual novelty and fluidity to the debate, to date it has had relatively little to say on issues of more foundational socio‐technical change beyond the urban sphere. In addition to introducing explicitly relational scalar sensibilities into this line of thinking, we have also aimed to emphasise the inherently disruptive, dissensual and materially hybrid characteristics of trans‐local low‐carbon transformations. Moving beyond the traditional lynchpin of emphasising socio‐technical innovation and market take‐up in sustainability experiments, we have also highlighted that relational rescaling is both strategic and messy, involving transformations in household‐level practices and new governance configurations alike.

An improved conceptualisation of scale can also benefit understandings of transformational change within Human Geography itself, by foregrounding how a variety of agencies are involved in allowing relatively localised interventions – or elements thereof – to travel from one place to another, or become implemented across a wider variety of territorial and governance contexts. There are lessons here about how geographers understand the transferability of knowledge and policy instruments within the context of low‐carbon interventions (Bulkeley, Castán Broto & Edwards, 2014). An interactionist view of the mobility of LCUIs within wider social contexts could mean that “as actors become more connected within networks, their position in the landscape becomes entrenched” (Inkpen et al., 2007, p. 547), subsequently allowing the “entrenched actors” to forge of new networks and connections across multiple scales. Ergo, a key challenge is developing “complex interrelationships and hybrid responses” (Hodson & Marvin, 2016, p. 17) against the background of a “precarious fragility of alternatives” (2016, p. 17) to the dominant social order.

The mutable nature of energy and carbon circulations means that they are deeply embedded in the socio‐environmental particularities of place, which places into doubt the extent to which identical LCUIs may be easily replicated across different geographical contexts without taking into account locationally‐specific cultural, political or technical circumstances. Future research agendas, therefore, could focus on how the rescaling of low‐carbon urban action both incorporates and overcomes such contradictions, while shedding further light on the ability of LCUIs to generate more‐than‐human political ecologies that both negotiate and extend beyond the entanglements of place (Robertson, 2018). The mobilisation of Human Geography can help move the mainstream literature on rescaling low‐carbon action beyond the triad of “hierarchical,” “vertical” and “horizontal” scaffolds (Fuhr et al., 2018) among seemingly passive spatial sites.

In positioning cities as active agents in the process of low‐carbon development, we have highlighted the role of hybrid socio‐technical networks in allowing knowledge and power to both move and be transformed in relation to relevant actors. In this, we reaffirm Castán Broto's (2015) view that the inherent contradictions contained in energy and climate‐related urban action may not themselves present an obstacle to further diffusion, but rather aid a process of active negotiation and reflection that helps build further connections and initiatives. A key political and practical challenge, however, is how to “monitor, document and take stock of dissensus‐driven practices and methods” (Kaika, 2017, p. 9) in the context of LCUIs as well as urban climate action more broadly. Rescaling can thus become a process driven by disruption and contestation within the social and built fabric of cities, as opposed to a technocratic activity of infrastructural and regulatory adjustment. From an emancipatory point of view, the theoretical corollary of this in Human Geography is the need to incorporate the contradictions of socio‐technical hybridisation and standardisation, while rupturing established injustice‐generating positions and practices.
